# Effect of Friction Stir Processing Parameters on the Surface Wear Resistance of 65Mn Steel

**DOI:** 10.3390/ma19142958

**Published:** 2026-07-09

**Authors:** Di Jiang, Hongfeng Wang, Bokai Jiao, Shuo Sun, Xianyi Zhou, Houzhen Ding, Xiuhui Cai

**Affiliations:** College of Mechanical and Electrical Engineering, Huangshan University, Huangshan 245000, China

**Keywords:** 65Mn steel, friction stir processing, microstructure, hardness, wear resistance

## Abstract

Friction stir processing (FSP) was applied to rolled 65Mn steel to improve its surface wear resistance, with untreated and conventionally water-quenched specimens used as reference conditions. A 3 × 3 full-factorial combination of rotational speeds of 600, 700, and 800 rpm and traverse speeds of 200, 250, and 300 mm/min was investigated. Continuous modified layers without obvious macroscopic defects were obtained under all processing conditions. FSP substantially fragmented and reconstructed the original ferrite–pearlite microstructure, producing a fine and relatively homogeneous acicular/lath-like transformed microstructure. The average grain size decreased from 27.1 μm to 11.6–17.2 μm, corresponding to reductions of 36.5–57.2%, while the high-angle grain-boundary fraction increased from 76.7% to a maximum of 86.2%. Although the hardness of the FSP-treated regions was comparable to that of the quenched specimen, FSP produced a more continuous and spatially uniform gradient-hardened layer. The minimum wear rate was obtained at 800 rpm and 200 mm/min, representing reductions of 97.34% and 96.14% relative to the base material and quenched specimen, respectively. The condition of 800 rpm and 300 mm/min was identified as the optimum overall condition because it provided a favorable balance among wear rate, mass loss, hardened-layer continuity, and wear-track morphology, with wear-rate reductions of 96.57% and 95.02% and mass-loss reductions of 66.67% and 57.14% relative to the two reference conditions. The improved wear resistance is primarily associated with grain refinement, grain-boundary reconstruction, microstructural homogenization, and the formation of a continuous gradient-hardened layer.

## 1. Introduction

65Mn steel is a typical high-carbon manganese spring steel that combines high strength, good elasticity, and low cost. It has been widely used in elastic components, cutting tools, and wear-resistant parts for agricultural and mining machinery. However, under complex friction and wear conditions, 65Mn steel is prone to severe surface damage, and its service life largely depends on the microstructural state and strengthening effect of the surface layer [1,2]. Therefore, effective regulation of the surface microstructure of 65Mn steel and improvement of its friction and wear performance are key scientific issues for extending its service life.

In terms of surface strengthening technologies, heat treatment, nitriding/carburizing, laser cladding, laser quenching, and thermal spraying have all been applied to improve the surface properties of 65Mn steel, and certain research progress has been achieved. Tong et al. [3] refined the microstructure of 65Mn steel through cyclic heat treatment, significantly improving its hardness and wear resistance; however, the strengthening effect was highly sensitive to the cyclic temperature and number of cycles. Lu et al. [4] markedly enhanced the tribocorrosion and wear resistance of 65Mn steel by plasma nitriding, although the improvement was strongly affected by the nitrided layer thickness and service environment. Wu et al. [5] and Yu et al. [6] prepared Fe-based gradient laser-cladded layers and multilayer Fe901 coatings, respectively, demonstrating that the introduction of hard phases can effectively improve surface hardness and wear resistance. Nevertheless, both approaches still face common challenges, including the reliability of the cladding interface and residual stress in the heat-affected zone. Liu et al. [7] obtained a refined martensitic microstructure by high-power, high-speed laser quenching, which reduced wear depth and increased microhardness, but the strengthening effect was highly dependent on the depth of the hardened layer. He et al. [8] also achieved improved hardness and wear behavior using pulsed detonation plasma technology. Overall, the above methods can improve the surface properties of 65Mn steel to varying degrees. However, they generally suffer from several limitations, such as high sensitivity to processing parameters, difficulty in microstructural control, the risk of brittle layers or interfacial defects, and relatively high processing costs. In particular, methods involving melting and solidification are more likely to induce defects such as pores, cracks, and residual stress concentration. Therefore, it is of great engineering significance to develop a solid-state surface modification method that combines microstructural refinement with gradient strengthening.

Friction stir processing (FSP), derived from friction stir welding (FSW), is a typical solid-state thermo-mechanically coupled surface modification technique. Its basic principle is to impose severe plastic deformation and frictional heat input on the near-surface region of a material through a rotating tool, thereby promoting material flow, microstructural fragmentation, dynamic recrystallization, and local homogenization. As a result, surface microstructure regulation and property enhancement can be achieved without macroscopic melting. Since the concept of FSP was first proposed by Mishra et al., numerous studies have confirmed that this technique offers significant advantages, including the avoidance of solidification defects, improved microstructural uniformity, and the ability to tailor local properties [9,10,11]. Therefore, FSP has gradually become an important research direction for surface strengthening of metallic materials.

In recent years, FSP has been increasingly applied to the surface modification of ferrous materials, including high-carbon steels, stainless steels, tool steels, high-speed steels, tempered martensitic steels, and wear-resistant rail steels. Pan et al. [12] reported that the traverse speed significantly affected the retained austenite content, carbide distribution, and surface hardness of FSP-treated AISI 440C high-carbon martensitic stainless steel. Aldajah et al. [13] applied FSP to AISI 1080 high-carbon steel and found that the thermomechanically induced microstructural transformation and surface hardening markedly improved its sliding wear resistance. Wu et al. [14] produced an ultrafine duplex microstructure through underwater FSP, resulting in simultaneous improvements in wear and corrosion resistance.

Previous reviews by Ralls et al. [15] and Merah et al. [16] demonstrated that the improvement in the surface properties of steels after FSP is generally related to the combined effects of severe plastic deformation, grain refinement, dynamic-recrystallization-assisted microstructural evolution, phase transformation, and surface hardening. They also emphasized that rotational speed, traverse speed, tool material, cooling conditions, and processing strategy strongly affect the final microstructure and performance of the processed layer.

More recently, Liu et al. [17] investigated multiple-pass FSP of M2 high-speed steel and revealed that the overlap ratio and subsequent thermal exposure significantly affected the microstructure, hardness, wear resistance, and corrosion behavior of the processed region. Falekari et al. [18] showed that FSP promoted grain refinement and increased the fraction of high-angle grain boundaries in thick tempered martensitic steel, thereby improving its hardness and mechanical properties. Shi et al. [19] further demonstrated that FSP can coordinately regulate the microstructure, hardness distribution, and wear behavior of U75V rail steel.

Collectively, these studies confirm the potential of FSP for strengthening ferrous materials. However, most previous studies have focused on stainless steels, tool steels, high-speed steels, or rail steels, whereas systematic investigations of 65Mn high-carbon manganese steel remain limited. In particular, the relationships among processing parameters, grain and grain-boundary evolution, hardness-layer continuity, and tribological performance have not yet been fully clarified. Moreover, direct comparisons between FSP-treated 65Mn steel and conventionally quenched steel under comparable hardness levels remain scarce.

## 2. Materials and Methods

**Figure 1:**
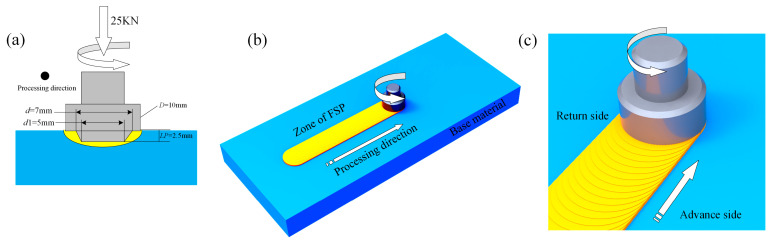
Schematic diagram of the FSP process. (**a**) Schematic diagram of the stirring tool dimensions and applied load. (**b**) Schematic diagram of the FSP process. (**c**) Schematic diagram of the FSP processing direction.

In this study, processing was performed using a stirring tool with a shoulder diameter of 10 mm and a conical pin length of 2.5 mm. The maximum and minimum diameters of the pin were 7 mm and 5 mm, respectively, and the tool material was tungsten carbide. The processing path was linear. The side on which the tool rotation direction was consistent with the processing direction was defined as the advancing side, while the opposite side was defined as the retreating side. The corresponding schematic diagram of the FSP process is shown in Figure 1.

**Figure 2:**
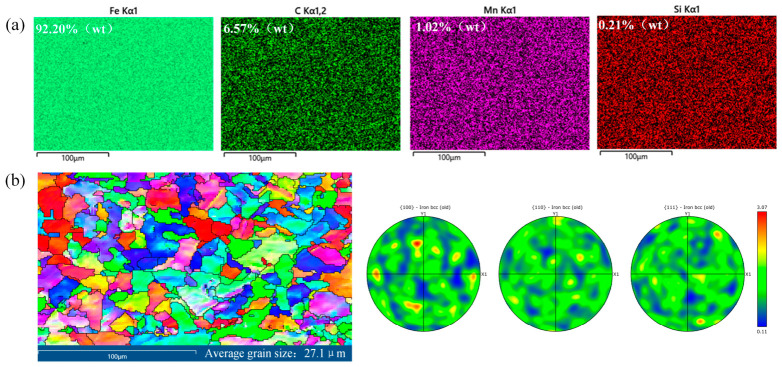
Qualitative EDS elemental maps and EBSD grain characteristics of the 65Mn base material. (**a**) Proportions of different elements in 65Mn steel; (**b**) EBSD image of 65Mn steel.

Table 1 presents the basic mechanical properties of the 65Mn base material, Table 2 lists the nominal bulk chemical composition of the 65Mn steel, nominal composition sup-plied by the manufacturer. whereas Figure 2 presents qualitative EDS elemental maps and EBSD grain characteristics of the base material. The elemental values displayed in the EDS maps were not used to determine the bulk chemical composition.

Since FSP is a technological extension of FSW, its processing parameters and tool characteristics are similar to those used in FSW. FSP was conducted using an FSW-A01 friction stir welding machine manufactured by Beijing Saifusite Co., Ltd., Beijing, China. Therefore, the processing parameters in this study were also selected based on previous studies. The rotational speeds were set to 600, 700, and 800 rpm, and the traverse speeds were set to 200, 250, and 300 mm/min. A full-factorial experimental design was adopted. The plunge depth was 0.1 mm, the axial force applied to the processing tool was 25 ± 0.3 kN, and the spindle tilt angle was 0°. The detailed processing parameters are listed in Table 3.

To support the relative comparison of the processing conditions, the rotational-speed-to-traverse-speed ratio, N/v, was calculated as a process index, where N is the rotational speed and v is the traverse speed. This parameter represents the number of tool revolutions per unit processing length and was used to characterize the relative thermomechanical severity of the different FSP conditions. The calculated N/v values ranged from 2.0 to 4.0 rev·mm^−1^. It should be emphasized that N/v is only a relative processing index and is not equivalent to the actual heat input, because the latter also depends on spindle torque, axial force, interfacial friction, material flow, and heat dissipation, which were not directly measured in the present study.

The metallographic specimens from the FSP region were ground using metallographic abrasive papers and then subjected to electrolytic polishing. Subsequently, the specimens were etched with a 4% nitric acid alcohol solution for 10 s and observed using an optical microscope. The FSP regions processed under different parameters were all polished to a mirror-like finish before characterization.

Vickers hardness testing was performed in accordance with ASTM E384-22 [20] using a pyramidal diamond indenter under a load of 1 kgf and a dwell time of 10 s. Hardness tests were performed in triplicate for each material condition, and the results are reported as the mean ± standard deviation.

The friction and wear tests were conducted in accordance with ASTM G99-23 [21] using a ball-on-disk configuration. A Si_3_N_4_ ball with a diameter of 4 mm was used as the counter body. The tests were performed under a normal load of 20 N along a circular wear track with a diameter of 4 mm at a rotational speed of 500 rpm for 20 min. Three independent wear tests were conducted for each material condition, and the wear rate, mass loss, and coefficient of friction are reported as the mean ± standard deviation. The wear volume and wear-track depth were measured using a Keyence three-dimensional ultra-depth-of-field microscope, and the worn surface morphologies were characterized using a Hitachi scanning electron microscope.

The EBSD data were processed using AZtecCrystal software version 2.1 (Oxford Instruments, High Wycombe, UK). The scanning step size was 0.25 μm. The EBSD scan area was 50 × 30 μm^2^, data cleaning was performed using a nearest-neighbor correlation procedure based on five adjacent points, with a maximum of 10 iterations. A misorientation angle of 15° was used as the threshold to distinguish low-angle grain boundaries from high-angle grain boundaries. Because AZtecCrystal does not employ the EDAX-defined confidence index, no confidence-index threshold was applied.

Meanwhile, a commonly used heat-treatment process was adopted as the control condition. The specimens were quenched at 850 ± 2 °C, held for 30 min, and then rapidly cooled in water at 25 ± 2 °C.

## 3. Results and Discussion

### 3.1. Macroscopic Characteristics and Microstructural Evolution

**Figure 3:**
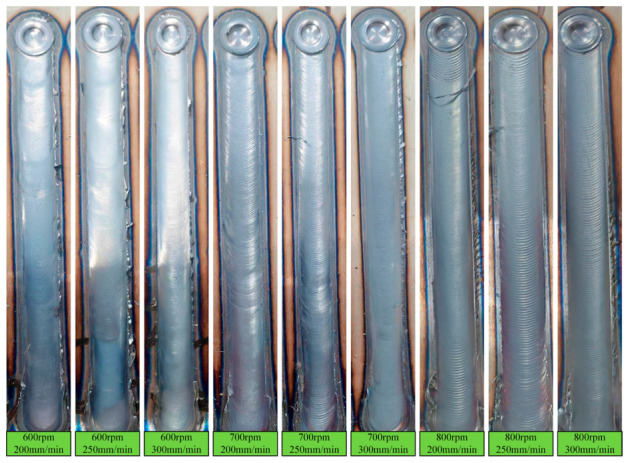
Macroscopic morphology of the FSP regions under different processing parameters.

Figure 3 shows the macroscopic morphology of the FSP regions produced under different processing parameters. From the surface appearance, all parameter combinations investigated in this study were able to produce FSP regions without obvious defects. At the relatively low rotational speed of 600 rpm, the most pronounced continuous burrs were formed on the advancing side of the FSP region. In contrast, at the highest rotational speed of 800 rpm, only a small amount of burr formation was observed in the first half of the processing path. This is attributed to the increased relative thermomechanical input and improved material plasticization associated with the higher rotational speed. Because the processing temperature was not directly measured, no quantitative conclusion is drawn regarding the local temperature distribution between the advancing and retreating sides.

**Figure 4:**
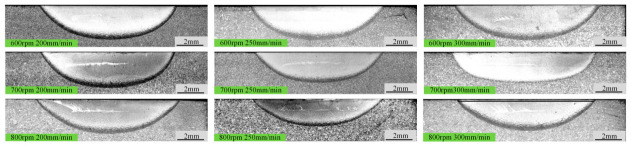
Cross-sectional morphology of the FSP regions under different processing parameters.

**Figure 5:**
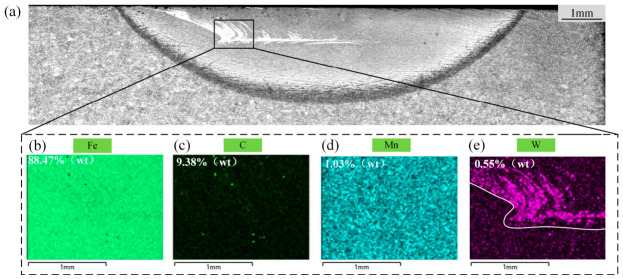
Cross-sectional elemental distribution of the FSP region. (**a**) Cross-sectional microstructure of the FSP region; elemental distribution maps of (**b**) Fe, (**c**) C, (**d**) Mn, and (**e**) W.

To further investigate the internal characteristics of the FSP zone, Figure 4 shows the macroscopic cross-sectional microstructures of the FSP regions under different processing parameters. It can be seen that no obvious internal defects are present in the FSP regions under any of the processing conditions. The FSP cross-section exhibits a semi-elliptical shape, with its maximum width being essentially consistent with the shoulder diameter of the tool, while its maximum depth reaches 2.6 mm, which is slightly greater than the pin length. This is mainly attributed to the fact that, during processing, the stirring tool induces plastic deformation in the surrounding material and generates frictional heat. In addition, a relatively distinct white band-like feature can be observed in the middle of the advancing side of the FSP cross-section, extending throughout the entire advancing side. A more detailed view of this region is provided in Figure 5.

As shown in Figure 5, the profile of the white band-like feature observed in the FSP cross-section is highly similar to the distribution profile of tungsten, as shown in Figure 5e. Meanwhile, the carbon content in this region is higher than that in the base material, as shown in Figure 5c, whereas the distribution and content of manganese show no obvious change. Considering that the stirring tool was made of tungsten carbide, the W- and C-rich band is inferred to originate from tool-wear debris detached during FSP and subsequently transported into the plasticized region of the 65Mn steel.

On the advancing side, the higher relative sliding velocity between the tool and the workpiece may promote localized tool wear and the detachment of W- and C-rich debris. The plastic flow around the rotating tool may subsequently transport this debris from the upper surface toward the interior of the processed region. This may explain why the W- and C-rich band is mainly located on the advancing side. However, the exact phase constitution of the incorporated material cannot be determined from the present elemental maps.

**Figure 6:**
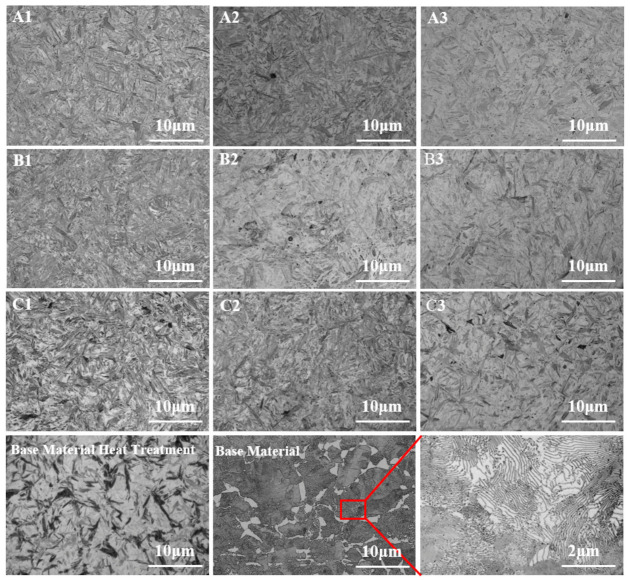
Metallographic microstructures of the materials. The labels (**A1**–**C3**) correspond to the processing parameters listed in Table 3.

Figure 6 shows the microstructural morphologies of the FSP regions in 65Mn steel under different processing parameters. Compared with the typical ferrite–pearlite microstructure of the original base material, the original lamellar pearlite is largely fragmented after FSP and transformed into a finer, denser, and more uniformly distributed acicular/bundle-like transformed microstructure. This indicates that significant microstructural reconstruction occurs under the coupled effects of severe plastic deformation and frictional heat. Overall, specimens A1–C3 no longer retain the clearly defined pearlite colonies or continuous ferrite networks observed in the base material. Instead, the microstructure is dominated by fine acicular and bundle-like features, demonstrating the pronounced grain-refining effect of FSP on the surface layer of 65Mn steel. Further comparison of the microstructures obtained under different parameters shows that, at lower rotational speeds, the stir zone exhibits a finer and more homogeneous microstructure. As the rotational speed increases, the acicular/lath-like features gradually become coarser, and the amount of local dark strip-like constituents increases. This suggests that the increased relative thermomechanical input facilitates material plasticization, microstructural reconstruction, and thermally activated grain-boundary migration, while also promoting local microstructural coarsening.

At the same rotational speed, increasing the traverse speed decreases the N/v value and shortens the tool–material interaction time per unit processing length, leading to a certain degree of overall microstructural refinement. As a result, the acicular/bundle-like microstructure becomes more uniformly distributed, and coarsening is suppressed. In summary, FSP significantly improves the coarse lamellar microstructure of the original 65Mn base material and produces a refined transformed microstructure. Among the investigated parameters, a lower rotational speed combined with a higher traverse speed is more favorable for obtaining a fine and uniform microstructure in the stir zone.

The microstructural evolution during FSP is governed by the combined effects of severe shear deformation, frictional heating, and rapid heat dissipation into the surrounding substrate. Increasing the rotational speed generally increases the relative thermomechanical input and improves material plasticization, whereas increasing the traverse speed shortens the interaction time and reduces the thermal exposure per unit length. Therefore, the final microstructure is determined by competition among deformation-induced fragmentation, thermally activated recovery and recrystallization, and subsequent microstructural coarsening. At relatively low rotational speeds, strong deformation and limited thermal exposure favor the retention of a relatively fine microstructure, although insufficient plasticization may reduce the continuity of the modified layer. At higher rotational speeds, enhanced thermal activation promotes material flow and microstructural reconstruction, but prolonged thermal exposure may also facilitate local coarsening.

The acicular/lath-like constituents observed in both the quenched and FSP-treated specimens indicate that thermally induced phase transformation may have occurred. During FSP, the combined action of frictional heating, severe plastic deformation, and rapid cooling through the surrounding material may transform the original ferrite–pearlite structure into a refined transformed microstructure. However, optical microscopy alone cannot unambiguously distinguish bainite, martensite, tempered martensite, or other transformation products. Therefore, the observed structure is described here as an acicular/lath-like transformed microstructure, and transformation-induced strengthening is considered a possible contribution rather than a conclusively identified mechanism.

**Figure 7:**
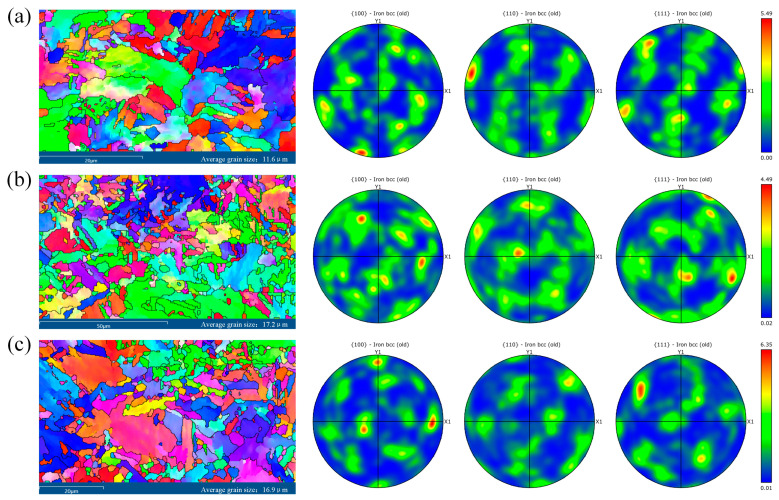
EBSD inverse pole figure maps and corresponding pole figures of the FSP regions. (**a**) 600 rpm, 300 mm/min; (**b**) 700 rpm, 300 mm/min; and (**c**) 800 rpm, 300 mm/min.

**Figure 8:**
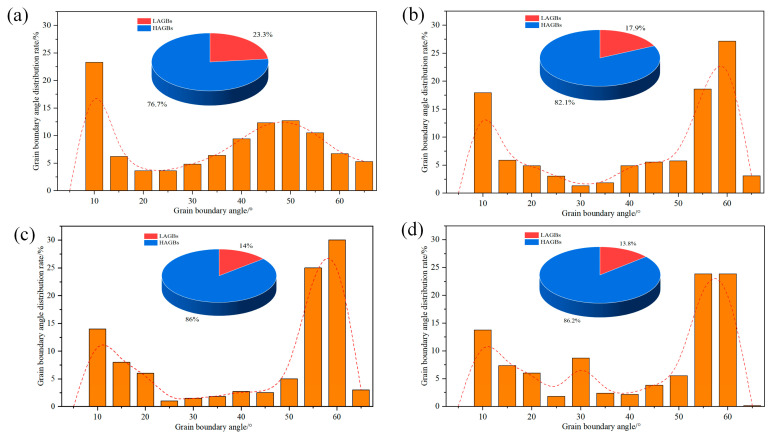
Grain boundary misorientation angle distribution in the FSP regions. (**a**) Base Material; (**b**) 600 rpm, 300 mm/min; (**c**) 700 rpm, 300 mm/min; (**d**) 800 rpm, 300 mm/min.

The EBSD results are consistent with the microstructural evolution described above. The average grain sizes in the stir zones under different FSP parameters are approximately 11.6 μm, 17.2 μm, and 16.9 μm, respectively, all of which are significantly smaller than that of the original base material, 27.1 μm. This indicates that FSP has a pronounced grain refinement effect on 65Mn steel. Finer grains are more readily obtained at lower rotational speeds. With increasing rotational speed and relative thermomechanical input, thermally activated recovery, grain-boundary migration, and microstructural reconstruction are promoted; however, grain growth may also be accelerated, which explains the larger grain sizes obtained at higher rotational speeds, as shown in Figure 7. Meanwhile, the FSP process also induces significant reconstruction of the grain boundary structure. Compared with the base material, the fraction of high-angle grain boundaries (HAGBs) in the FSP region increases markedly and becomes more pronounced with increasing rotational speed. Under the condition of 800 rpm, the HAGB fraction increases from 76.7% in the base material to 86.2%, as shown in Figure 8d. This suggests that the material undergoes severe shear deformation, subgrain formation, grain boundary migration, and dynamic recrystallization during FSP. As a result, the original grain-boundary network is progressively reconstructed into a refined structure containing a higher fraction of high-angle grain boundaries.

During FSP, the intense shear deformation imposed by the rotating tool causes rapid dislocation multiplication and accumulation within the original grains. With continued deformation, the dislocations rearrange into dislocation cells and subgrain boundaries, producing a progressively subdivided microstructure. Under the assistance of frictional heating, recovery, subgrain rotation, and grain-boundary migration may increase the misorientation across these subboundaries. Consequently, some deformation-induced low-angle grain boundaries gradually evolve into high-angle grain boundaries, resulting in the formation of refined grains with more stable boundary configurations.

The reduction in average grain size from 27.1 μm in the base material to approximately 11.6–17.2 μm in the representative FSP regions, together with the increase in the high-angle grain-boundary fraction from 76.7% to a maximum of 86.2%, is consistent with dynamic-recrystallization-assisted grain-boundary reconstruction. Nevertheless, the available EBSD results do not allow an unambiguous distinction between continuous and discontinuous dynamic recrystallization. Therefore, the microstructural evolution is interpreted as being associated with dynamic recrystallization rather than being assigned to a single specific recrystallization mechanism.

Compared with the base material, the representative FSP conditions reduced the average grain size by approximately 36.5–57.2%, while the HAGB fraction increased by up to 9.5 percentage points. These results quantitatively demonstrate the pronounced grain-refinement and grain-boundary-reconstruction effects of FSP. Because EBSD measurements were available for only three representative FSP conditions, the variations in grain size and HAGB fraction are interpreted as representative microstructural trends rather than statistically conclusive correlations.

The texture intensity also varies among the FSP conditions, indicating that the crystallographic orientation distribution is sensitive to the processing parameters. The observed texture evolution can be attributed to the combined effects of shear deformation, material flow, grain-boundary migration, and thermally activated microstructural reconstruction. However, texture intensity alone is insufficient to determine the degree of recrystallization. Therefore, the texture results are used primarily to demonstrate the influence of the relative thermomechanical input and plastic-flow conditions on crystallographic reorientation during FSP.

### 3.2. Hardness Distribution and Strengthening Behavior

**Figure 9:**
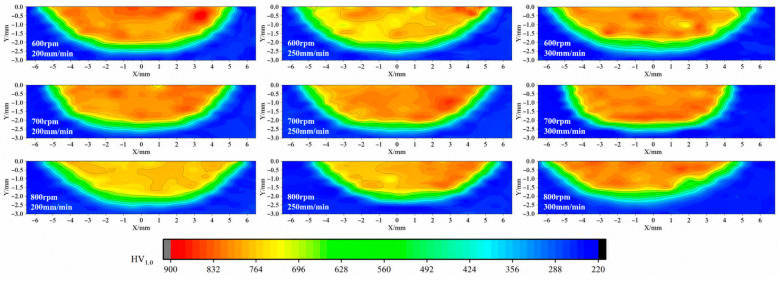
Hardness distribution contour maps of the FSP regions.

Figure 9 presents the hardness contour maps under different processing parameters. These results further validate the microstructural evolution described above. Under all parameter conditions, the FSP cross-sections exhibit a semi-elliptical high-hardness region that generally matches the geometric profile of the stir zone, indicating that FSP can produce a pronounced gradient-strengthening effect. At the relatively low rotational speed of 600 rpm, the distribution of the high-hardness region shows noticeable fluctuations, and the extent of the high-hardness zone becomes narrower as the traverse speed increases. This suggests that, under low rotational-speed conditions, the degree of material plasticization is limited and the continuity of the hardened layer is insufficient. At a rotational speed of 700 rpm, the hardened layer expands significantly, and when the traverse speed is 250 mm/min, the high-hardness region is the widest and the hardness distribution is the most concentrated, indicating that under this parameter combination, the combined stirring action and relative thermomechanical input can promote possible transformation-induced strengthening and microstructural refinement. When the rotational speed is increased to 800 rpm, the high-hardness region becomes more uniformly distributed. In particular, at a traverse speed of 300 mm/min, the high-hardness region shows the closest correspondence with the contour of the stir zone, indicating that a continuous and stable strengthened layer is formed under this condition. Overall, the hardness-strengthened layer produced by FSP does not depend solely on a local peak hardness, but rather on the continuity and width of the high-hardness region, as well as its gradient transition along the thickness direction. Therefore, a uniform and continuous hardened layer is more beneficial for load transfer and wear suppression during subsequent service.

When the hardness results are considered together with the EBSD observations, the formation of the high-hardness region is closely associated with grain refinement and grain-boundary reconstruction. Compared with the base material, the representative FSP conditions reduced the average grain size from 27.1 μm to approximately 11.6–17.2 μm, corresponding to reductions of 36.5–57.2%, while the HAGB fraction increased from 76.7% to a maximum of 86.2%. The increased grain-boundary density provides additional barriers to dislocation motion and therefore contributes to the enhanced resistance to plastic deformation. However, the comparable hardness levels of the FSP-treated and quenched specimens indicate that grain refinement alone cannot fully account for the measured hardness. Possible contributions from the transformed acicular/lath-like microstructure, deformation-induced dislocation strengthening, and the continuity of the gradient-hardened layer should also be considered.

The grain refinement induced by FSP contributes to the hardness enhancement through the Hall–Petch effect, which can be expressed in a hardness-related form as H = H_0_ + kHd^−1/2^, where H is the hardness, H_0_ is the intrinsic hardness, kH is the Hall–Petch coefficient, and d is the average grain size. The reduction in average grain size from 27.1 μm in the base material to approximately 11.6–17.2 μm in the representative FSP regions increased the d^−1/2^ term by approximately 25.5–52.8%. The increased grain-boundary density provides more barriers to dislocation motion and therefore improves the resistance of the processed surface to plastic deformation and indentation.

However, the hardness distribution cannot be explained solely by grain refinement. The acicular/lath-like transformed microstructure indicates that possible transformation-induced strengthening may also contribute to the high hardness of the processed region. In addition, severe plastic deformation during FSP can generate a high dislocation density, further increasing resistance to plastic deformation. Since the FSP-treated and conventionally quenched specimens exhibited generally comparable hardness levels, the principal advantage of FSP is not necessarily a further increase in peak hardness, but the formation of a more continuous, spatially uniform, and gradually distributed hardened layer.

The W- and C-rich band observed on the advancing side is spatially associated with the white band-like feature identified in the processed region. Since the stirring tool was manufactured from tungsten carbide, this enrichment is inferred to originate from tool-wear debris detached during FSP and transported into the plasticized steel. However, the present elemental mapping results cannot determine whether the incorporated material remains as intact WC particles, undergoes fragmentation or partial dissolution, or forms other W-containing constituents.

Moreover, local hardness measurements were not performed separately inside and outside the W- and C-rich band, and the volume fraction and spatial distribution of the enriched material were not quantitatively determined. Therefore, its specific contribution to the hardness and wear resistance cannot be isolated from those of grain refinement, microstructural transformation, grain-boundary reconstruction, and gradient hardening. The role of the W- and C-rich band should consequently be regarded as unresolved in the present study. Quantitative assessment will require local microhardness or nanoindentation measurements, phase-level characterization, and controlled comparisons under conditions with different levels of tool wear.

### 3.3. Tribological Performance and Worn-Surface Characteristics

**Figure 10:**
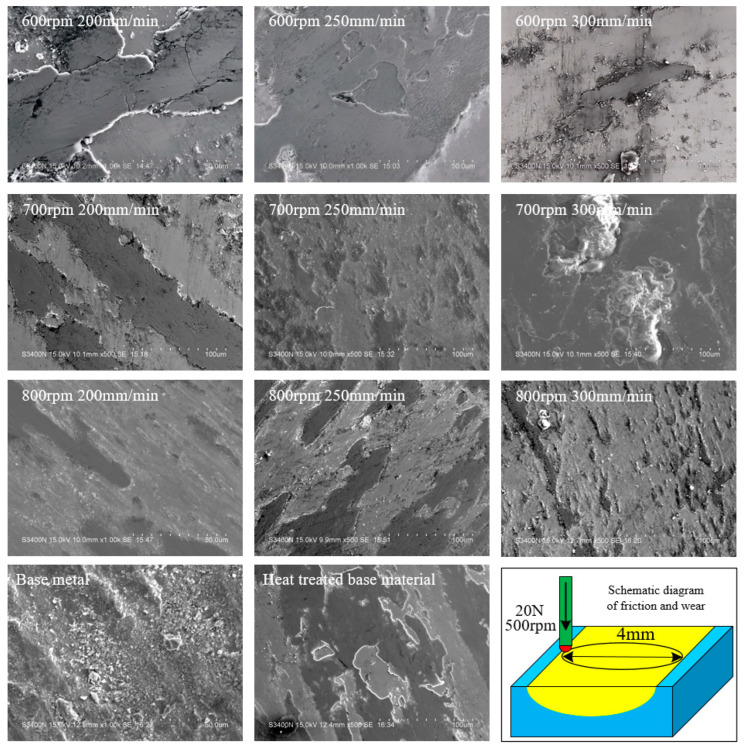
SEM morphologies of the worn surfaces in the FSP regions.

**Figure 11:**
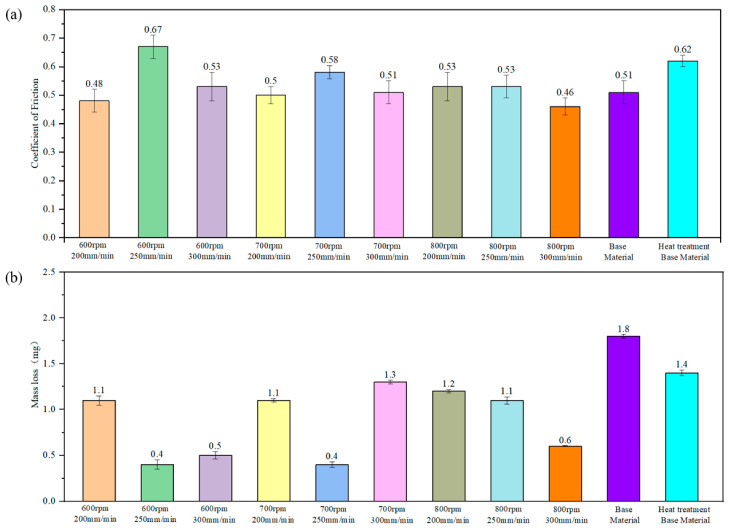
Friction coefficient and wear loss of the FSP regions (the error bars represent the standard deviation of three tests). (**a**) Coefficient of friction; (**b**) Mass loss.

**Figure 12:**
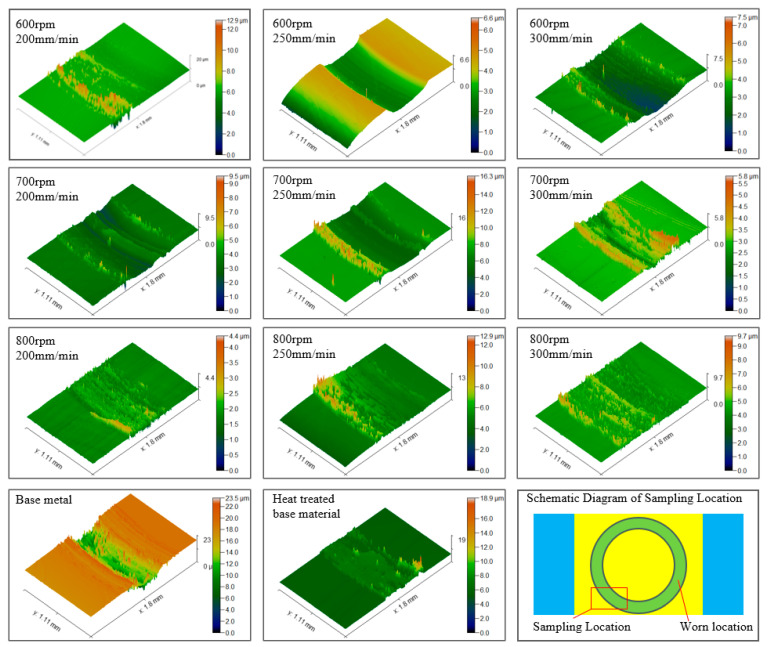
Three-dimensional worn surface morphologies of the FSP regions (for qualitative morphological comparison only).

**Figure 13:**
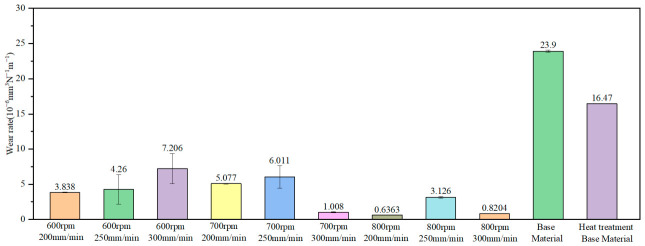
Wear rate distribution of the friction and wear regions in the FSP zones (the error bars represent the standard deviation of three tests).

Figure 10, Figure 11, Figure 12 and Figure 13 show the friction coefficients, mass losses, worn surface morphologies, three-dimensional wear-track maps, and wear-rate distributions of the FSP regions, the base material, and the heat-treated base material, respectively. The three-dimensional wear-track maps were used to qualitatively compare the wear-track morphology, relative depth, material accumulation, and localized spalling among the investigated conditions. Since standard areal roughness parameters and directional descriptors were not extracted from the original topographical datasets, these maps should not be interpreted as a quantitative evaluation of surface roughness or wear-track anisotropy.

The friction and wear results are in good agreement with the microstructural and hardness characteristics. Overall, the wear rate and mass loss of the FSP specimens are significantly lower than those of both the original base material and the heat-treated base material, indicating that FSP markedly improves the wear resistance of 65Mn steel. The original base material exhibits the highest wear rate and mass loss, followed by the heat-treated base material. This suggests that although heat treatment alone can increase the hardness of the material, it is still difficult to effectively suppress material removal and surface spalling during friction because of the relatively coarse and non-uniform microstructure, as shown in Figure 11 and Figure 13. In contrast, the wear rates of the FSP specimens are generally substantially reduced, indicating that their improved wear resistance mainly originates from the enhanced surface load-bearing capacity provided by the refined and homogeneous strengthened surface microstructure. It is worth noting that the friction coefficient varies only slightly among different FSP specimens, whereas the differences in wear rate and mass loss are much more pronounced. This indicates that the improvement in wear resistance is not primarily determined by a reduction in the friction coefficient, but is more closely related to the reduced material removal and lower degree of surface damage during the wear process.

By combining the wear rate, mass loss, three-dimensional wear track morphology, and SEM characteristics under different processing parameters, the parameter sensitivity of the friction and wear behavior can be further clarified. At a rotational speed of 600 rpm, the specimens processed at traverse speeds of 200 and 250 mm/min exhibit relatively shallow wear tracks, with the worn surfaces mainly characterized by slight grooves and localized micro-spalling. The corresponding wear mechanism is still dominated by mild abrasive wear, as shown in Figure 10. However, when the traverse speed is increased to 300 mm/min, the wear rate increases markedly, and surface debris and localized tearing become more pronounced. This indicates that increasing the traverse speed at a low rotational speed weakens the stability of the surface microstructure and strengthened layer, thereby intensifying material removal.

At a rotational speed of 700 rpm, the specimens processed at traverse speeds of 200 and 250 mm/min show more obvious plastic flow and localized adhesive features. The wear mechanism gradually changes from simple abrasive wear to a combination of adhesive wear and fatigue spalling. Similar material-transfer behavior has been reported in sliding tribological contacts, where detached material adheres to the counter body or worn surface and subsequently alters the local contact conditions [22]. Milojević et al. [22] showed that the formation of a transferred layer was closely associated with adhesive wear and fluctuations in the frictional response, supporting the interpretation of the adhesive features observed in the present study. When the traverse speed is increased to 300 mm/min, the wear rate of the specimen decreases to a relatively low level, but the mass loss remains relatively high. Combined with the SEM observations, an obvious transfer layer and localized spalling can be observed on the worn surface, indicating that although volumetric wear is suppressed under this parameter condition, flake-like spalling and local delamination remain relatively prominent.

When the rotational speed is increased to 800 rpm, the FSP-treated specimens exhibit the most favorable overall tribological performance among the investigated rotational-speed groups. At a traverse speed of 200 mm/min, the specimen shows the minimum wear rate, indicating the strongest resistance to volumetric material removal. However, its relatively high mass loss and localized spalling suggest that the wear behavior cannot be evaluated solely from the wear rate. When the traverse speed is increased to 300 mm/min, the specimen exhibits a slightly higher wear rate but lower mass loss, a visually smoother wear-track morphology, less pronounced localized spalling, and a more continuous hardened layer. These results indicate that the two conditions provide different performance advantages.

In the present study, the optimum overall condition was not defined solely by the minimum wear rate. Instead, the wear rate, mass loss, wear-track morphology, localized spalling, and continuity of the hardened layer were considered together. Although the wear rate at 800 rpm and 300 mm/min was approximately 28.9% higher than that at 800 rpm and 200 mm/min, the former condition exhibited lower mass loss, less pronounced localized spalling, a visually smoother wear-track morphology, and a more continuous hardness distribution. Therefore, 800 rpm and 200 mm/min is identified as the minimum-wear-rate condition, whereas 800 rpm and 300 mm/min is identified as the optimum overall condition.

The N/v values ranged from 2.0 to 4.0 rev·mm^−1^. Among the investigated conditions, the highest N/v condition of 800 rpm and 200 mm/min produced the minimum wear rate, whereas the condition of 800 rpm and 300 mm/min, corresponding to a moderate N/v value of 2.7 rev·mm^−1^, provided a more favorable overall balance among material plasticization, relative thermal exposure, hardened-layer continuity, and resistance to localized spalling.

Considering the microstructural, hardness, and wear results together, the representative FSP conditions reduced the average grain size by approximately 36.5–57.2% and increased the HAGB fraction by up to 9.5 percentage points relative to the base material. These microstructural changes were accompanied by the formation of a broader and more continuous high-hardness region. Meanwhile, the minimum-wear-rate condition reduced the wear rate by 97.34% relative to the base material and by 96.14% relative to the quenched specimen, whereas the optimum overall condition produced corresponding reductions of 96.57% and 95.02%. These quantitative comparisons indicate that grain refinement and grain-boundary reconstruction contributed to improved resistance to plastic deformation and material removal.

However, the relationships among grain size, HAGB fraction, hardness, and wear rate were not strictly proportional. In particular, the FSP-treated and quenched specimens exhibited generally comparable hardness levels, whereas the FSP-treated specimens showed markedly lower wear rates and mass losses. Therefore, hardness alone cannot account for the observed tribological improvement. The continuity of the gradient-hardened layer, the uniformity of the transformed microstructure, and the suppression of localized delamination and fatigue spalling also contributed to the wear response.

Because EBSD measurements were available for only three representative FSP conditions, and the hardness results were obtained as spatial contour distributions rather than a single directly comparable scalar value for each condition, formal statistical correlations among grain size, HAGB fraction, hardness, and wear rate were not established. Accordingly, the relationships discussed above should be interpreted as quantitative comparative trends rather than statistically validated correlations.

In addition to hardness and microstructural characteristics, the directional distribution of grooves and the anisotropy of the worn surface may affect the local contact conditions and frictional response. Afferrante et al. [23] demonstrated that the anisotropic statistical properties of rough surfaces can alter contact mechanics and friction-related forces. Although their model was developed for viscoelastic rough contacts, it highlights the general importance of surface directionality in tribological interactions. In the present study, however, quantitative directional descriptors were not available; therefore, the possible influence of wear-track anisotropy is discussed only qualitatively.

### 3.4. Strengthening and Wear-Resistance Mechanisms

The improvement in the wear resistance of FSP-treated 65Mn steel originates from the synergistic effects of grain refinement, grain-boundary reconstruction, microstructural homogenization, possible transformation-induced strengthening, and the formation of a continuous gradient-hardened layer. During FSP, severe shear deformation fragments the original ferrite–pearlite structure and produces a high density of dislocations, dislocation cells, and subgrain boundaries. Thermally activated recovery, subgrain rotation, and grain-boundary migration subsequently promote dynamic-recrystallization-assisted grain refinement and increase the fraction of high-angle grain boundaries. The refined grains and reconstructed boundary network impede dislocation motion, thereby increasing the resistance of the surface layer to plastic deformation.

The possible formation of transformed acicular/lath-like constituents provides an additional contribution to hardness. However, because their exact phase constitution has not been directly identified, the transformation-related contribution is not assigned to a specific bainitic or martensitic mechanism. A W- and C-rich band was also detected in the processed region; however, its independent contribution to hardness and wear resistance remains unresolved because its local mechanical effect was not quantitatively characterized.

Although the hardness of the FSP-treated region is generally comparable to that of the conventionally quenched specimen, the FSP-treated specimens exhibit substantially lower wear rates and mass losses. This result demonstrates that wear resistance is not controlled solely by peak hardness. The quenched specimen contains a relatively coarse and less uniform transformed microstructure, which may produce localized stress concentration and facilitate crack initiation, delamination, and flake-like spalling. In comparison, FSP produces a refined and relatively homogeneous microstructure together with a continuous gradient transition from the hardened surface layer to the underlying substrate. This gradient structure improves load transfer, reduces severe localized deformation, and delays the initiation and propagation of subsurface cracks.

The relatively small differences in friction coefficient among the FSP-treated specimens, together with the pronounced differences in wear rate and mass loss, further indicate that the tribological improvement is primarily associated with the suppression of material removal rather than a substantial reduction in interfacial friction. Grain refinement and surface hardening reduce penetration and ploughing by the Si_3_N_4_ counter body, while the improved microstructural uniformity and hardened-layer continuity suppress plastic flow, delamination, and fatigue-induced spalling.

The effects of rotational speed and traverse speed are non-monotonic because the processing parameters simultaneously affect deformation intensity, material plasticization, thermal exposure, microstructural refinement, and hardened-layer continuity. At a relatively low rotational speed, insufficient thermal activation limits material flow and the continuity of the strengthened layer, although the microstructure may remain relatively fine. Increasing the rotational speed improves material plasticization and facilitates the formation of a broader and more continuous modified layer. Excessive thermal exposure, however, may promote local microstructural coarsening and adhesive deformation. Therefore, the optimum overall tribological performance is achieved through a balance among deformation-induced refinement, thermally activated microstructural reconstruction, and hardened-layer continuity.

The condition of 800 rpm and 200 mm/min was identified as the minimum-wear-rate condition, indicating the strongest resistance to volumetric material removal. However, its relatively high mass loss suggests that localized flake-like spalling remained significant. In contrast, the specimen processed at 800 rpm and 300 mm/min exhibited a slightly higher wear rate but lower mass loss, a visually smoother wear-track morphology, and a more continuous hardness distribution. Therefore, 800 rpm and 300 mm/min was identified as the optimum overall condition because it provided a favorable balance among wear rate, mass loss, hardened-layer continuity, wear-track morphology, and resistance to localized spalling.

Overall, the strengthening and wear-resistance mechanism can be summarized as follows: severe thermomechanical deformation induces fragmentation of the original microstructure and dynamic-recrystallization-assisted grain refinement; grain-boundary reconstruction, possible transformation-induced strengthening, and increased dislocation density improve resistance to plastic deformation; the continuous gradient-hardened layer enhances load transfer and suppresses subsurface damage; and the possible contribution of the W- and C-rich tool-wear debris remains unresolved because its local mechanical effect was not independently quantified.

Another limitation of the present study is that the three-dimensional wear-track maps were analyzed primarily from a morphological perspective. Quantitative areal roughness parameters and directional descriptors, including Sa, Sq, Sz, Str, Std, and Sal, were not determined. Consequently, the effects of groove orientation, ploughing direction, and roughness anisotropy on the friction coefficient and wear response could not be quantitatively separated from the effects of hardness and microstructure. Future work should combine wear-volume measurements with ISO 25178-2:2021-based areal-topography analysis to clarify the relationships among surface morphology, anisotropy, friction, and material removal [24].

## 4. Conclusions

In this study, friction stir processing was applied to the surface modification of rolled 65Mn steel, with the untreated base material and conventionally water-quenched specimens used as reference conditions. The effects of rotational speed and traverse speed on the forming quality, microstructural evolution, grain-boundary characteristics, hardness distribution, and tribological performance were systematically investigated. The following conclusions can be drawn:

(1) Within the investigated rotational-speed range of 600–800 rpm and traverse-speed range of 200–300 mm/min, continuous FSP-modified layers without obvious macroscopic defects were successfully produced. The processed region exhibited a semi-elliptical cross-sectional profile, with a maximum width approximately corresponding to the tool-shoulder diameter. FSP substantially fragmented and reconstructed the original ferrite–pearlite microstructure, producing a fine, dense, and relatively homogeneous acicular/lath-like transformed microstructure. Because direct phase-identification evidence was not available, this transformed microstructure was not assigned to a specific bainitic or martensitic constituent.
(2) FSP promoted pronounced grain refinement and grain-boundary reconstruction in the surface layer of 65Mn steel. The average grain size decreased from 27.1 μm in the base material to approximately 11.6–17.2 μm in the representative FSP regions, while the fraction of high-angle grain boundaries increased from 76.7% to a maximum of 86.2%. These changes are consistent with dynamic-recrystallization-assisted microstructural evolution during severe thermomechanical deformation. A continuous semi-elliptical gradient-hardened layer corresponding closely to the stir-zone profile was also formed. Although the hardness level of the FSP-treated region was generally comparable to that of the quenched specimen, FSP produced a more continuous and spatially uniform high-hardness region. The improved hardness and hardened-layer continuity are attributed primarily to the combined effects of grain refinement, grain-boundary reconstruction, microstructural homogenization, and possible transformation-induced strengthening.
(3) All FSP-treated specimens exhibited substantially lower wear rates and mass losses than the untreated base material and conventionally quenched specimen. The minimum wear rate was obtained at 800 rpm and 200 mm/min, with reductions of 97.34% and 96.14% relative to the base material and quenched specimen, respectively. At 800 rpm and 300 mm/min, the wear rate was reduced by 96.57% and 95.02%, while the mass loss was reduced by 66.67% and 57.14% relative to the two reference conditions, respectively. Although this condition did not produce the absolute minimum wear rate, it exhibited lower mass loss, less pronounced localized spalling, a visually smoother wear-track morphology, and a more continuous hardened layer. Therefore, 800 rpm and 200 mm/min was identified as the minimum-wear-rate condition, whereas 800 rpm and 300 mm/min was identified as the optimum overall condition.

## Figures and Tables

**Table 1:** Mechanical and physical properties of 65Mn.

Tensile Strength/MPa	Elongation/%	Hardness/HV_1.0_	Average Grain Size/μm
772	12	256	27.1

**Table 2:** Nominal chemical composition of 65Mn.

Fe	C	Mn	Si	P	Others
Balance	0.65%	1.1%	0.30%	0.03%	0.02%

**Table 3:** Processing parameter settings.

No.	Rotational Speed/rpm	Traverse Speed/mm·min^−1^	N/v rev·mm^−1^	Plunge Depth	Axial Force
A1	600	200	3.0	0.1 mm	25 ± 0.3 kN
A2	600	250	2.4		
A3	600	300	2.0		
B1	700	200	3.5		
B2	700	250	2.8		
B3	700	300	2.3		
C1	800	200	4.0		
C2	800	250	3.2		
C3	800	300	2.7		

## Data Availability

The original contributions presented in this study are included in the article. Further inquiries can be directed to the corresponding author.
